# The General Medical Council and the Supply of Doctors

**Published:** 1917-06-02

**Authors:** 


### The Hospital

The Workers' Newspaper of Administrative Medicine and Institutional Life, Administration, National Insurance and Health.

No. 1617, Vol. LXII. SATURDAY, JUNE 2, 1917. PRICE ONE PENNY

### THE GENERAL MEDICAL COUNCIL AND THE SUPPLY OF DOCTORS.

The meetings of the General Medical Council are not as a rule distinguished by the qualities which attract popular attention, and it may be doubted whether they stir more than a mere languid interest even among members of the profession. What the Council has to do is to carry into the sphere of practical administration the provisions of certain Acts of Parliament, and the exciting opportunities of what is commonly termed medico-political agitation are denied to it. It stands not for any class interest, but for the public welfare, as this has been defined by decisions of the Legislature, and it is from this point of view that its proceedings are to be judged. Critics of the Council, it is true, often demand activities outside this range, but such criticism only shows that its authors are ignorant of the essential facts of the situation. As a businesslike body addressing itself strictly to the responsibilities committed to its care the Council may readily find an abundant justification, and it has always steadily resisted the pressure of those who would urge it to more expansive enterprises. To say this is not to condemn such enterprises. These may, indeed, be of high value and wisdom. But the General Medical Council is not a suitable instrument for their execution. And its influence in the particular sphere it is called upon to occupy is due in no small measure to its abstinence from all things other than its proper business. The Council speaks with authority on the concerns committed to it, and it leaves other functions severely alone. Hence it discharges its duties with a minimum of friction, maintains a high standard of efficiency, and attracts the respect and confidence of the various Government Departments in touch with its activities. Its order of public service is not a dramatic one, but that it is of essential value in the complex life of the community there can be no question.

The recent meeting of the Council shares the interest which attaches itself at the present juncture to all proceedings concerned with the supply and distribution of medical practitioners. With the demands of the Army on the one hand, and the necessities of the civil population on the other, it has become a question how to make the best use of the existing numbers of practitioners. That the numbers cannot be increased is one of the hard facts of the situation, and hence arises the debate whether the supply as it exists is being wisely used. Like many others we had hoped that the Council could have afforded some information on this point, and all the more so seeing that the President of the Council had recently presided at a special conference summoned by the Director-General of National Service and charged with the study of this very question. Unhappily, all we can be told is that the members of this conference, representing the Professional Committees and certain Government Departments, have made definite proposals on the subject of a more effective distribution of our resources to the Director-General and that these proposals are under the consideration of the Government. It is something to know that the proposals were adopted unanimously by the conference, for the subject is a difficult and complex one and there have been manifest indications of a sharp difference of opinion in the profession. Thus when action comes there may be the need to ask for the subordination of personal views to official decisions taken after consideration of the best opinion available. It is significant in this respect that the President of the General Medical Council should say deliberately "the sources of supply [of medical practitioners] for service abroad and at home are in fact becoming severely straitened"; and should add as his personal opinion a doubt "whether, without fresh legislation, the present powers of the authorities concerned will suffice to cope with all the demands of the situation." What can be said on the whole position by the Council and by the Professional Committees has been said, and the next step rests with the Government. That the profession will give a patriotic response to any demand that may be advanced is certain. But it must be remembered that already much has been yielded, and that a full measure of goodwill can only be secured by fair and open dealing, and that arbitrary and autocratic action will defeat its own ends.

There are two aspects of the position on which the profession has a right to expect assurance. The one is that the inquiries which have been made have included a review not only of the distribution of practitioners in civil life, but also of those serving with the Army. Whether well-founded or not there is a widespread belief that the military medical authorities are not using to the best advantage all the men which the profession has given to them, and that many find that they have little or nothing to do. The second point which needs emphasis is that the question must be considered as a whole, and not split sharply into military and civil departments. More than ever the profession is one and must be dealt with in a manner which this proposition involves. We are yielding much to military claims for the sake of the national cause, but in their turn the military must recognise that the civilian status remains unimpaired even in the midst of an earth-shaking war.

